# A Novel Heterozygous Variant in F2 Gene in a Chinese Patient With Coronary Thrombosis and Acute Myocardial Infarction Leads to Antithrombin Resistance

**DOI:** 10.3389/fgene.2020.00184

**Published:** 2020-03-03

**Authors:** Yi Tang, Liyang Zhang, Wenlin Xie, Jieyuan Jin, Yujiao Luo, Mingyang Deng, Zhengyu Liu, Hong Wei Pan, Yi Zhang, Zhaofen Zheng, Liang-Liang Fan

**Affiliations:** ^1^Department of Cardiology, Hunan Provincial People’s Hospital, The First Affiliated Hospital of Hunan Normal University, Hunan Normal University, Changsha, China; ^2^Department of Neurosurgery, The XiangYa Hospital, Central South University, Changsha, China; ^3^Department of Pathology, The Seventh Affiliated Hospital of Sun Yat-sen University, Shenzhen, China; ^4^Department of Cell Biology, School of Life Sciences, Central South University, Changsha, China; ^5^Department of Hematopathology, The Second Xiangya Hospital, Central South University, Changsha, China; ^6^Hunan Key Laboratory of Animal for Human Disease, School of Life Sciences, Central South University, Changsha, China

**Keywords:** F2, coronary thrombosis, acute myocardial infarction, mutation, antithrombin resistance

## Abstract

Thrombophilia refers to a group of conditions where the blood clots more easily than normal. These blood clots can cause problems such as deep vein thrombosis or pulmonary embolism. Most kinds of mutated coagulation factors II (F2) exhibit lower procoagulant activity, but in some cases, a higher coagulation rate has been observed. The underlying mechanism is that those variations can prevent F2s from being inhibited by antithrombin, leading to a contiguous activation of procoagulation, and causing recurrent thromboembolism. In this study, a patient was admitted to our hospital due to repeated chest pain for 2 days and aggravated for 4 h. A medical history investigation showed that he had three deep venous thromboses in the lower limbs and one portal vein thrombosis events during the past 10 years. The electrocardiogram showed Q wave elevation and slight ST segment elevation in lead V2, and coronary angiogram showed a total occlusion of the left anterior descending artery. Laboratory testing found that troponin I was obviously elevated. Family history also indicated that both his father (II-3) and grandfather (I-1) died from pulmonary thromboembolism. Whole-exome sequencing was performed to detect the genetic lesion of the patient, and a novel mutation (c.1621 C>T/p.R541W) of *F2* was identified in the patient. This novel mutation resulted in a substitution of arginine by tryptophan, leading to antithrombin resistance (ATR). Our study is consistent with previously published papers. In conclusion, this study not only identifies a novel mutation of *F2* and will contribute to the genetic diagnosis and counseling of families with thrombosis but also suggests that the site p.R541 of *F2* may play a crucial role in thrombosis.

## Introduction

Thrombophilia is an increased tendency to form abnormal blood clots in blood vessels (Lim and Moll, 2015; Stevens et al., 2016; Skelley et al., 2017). Both inherited and acquired kinds, could play a significant role in the pathogenesis of deep vein thrombosis, pulmonary embolism, and arterial thrombotic disorders (Stevens et al., 2016). A large number of people with thrombophilia do not have symptoms and never have health problems (Castaman et al., 2019). Symptoms only occur if thrombophilia causes a blood clot (Connors, 2017).

Currently, many genetic factors have been identified in patients with thrombophilia. Mutations in *methylenetetrahydrofolate reductase* (*MTHFER*), *factor V Leiden* and *prothrombin* 20210 mutations were the main genetic lesions. In addition, protein S deficiency, protein C deficiency, antithrombin deficiency, dysfibrinogenemia and increased factor VIII have also been proven to lead to thrombosis formation (Stevens et al., 2016; Ahangari et al., 2019). According to a previous study of the Caucasian Australian population, 64.2% and 24.5% of the population were homozygous and heterozygous mutation carriers of four inherited thrombophilic mutations: Factor V Leiden (c.G1691A), prothrombin (c.G20210A), MTHFR (c.C677T and c.A1298C) (Gibson et al., 2005).

Coagulation factor II is proteolytically cleaved to form thrombin in the first step of the coagulation cascade which ultimately results in the stemming of blood loss. Thrombin has been characterized as an allosteric enzyme regulated by sodium binding, which is governed by five amino acid residues: Thr540, Arg541, Glu592, Arg596, and Lys599. Mutations identified at those residues may prevent F2 from being inhibited by antithrombin, which will lead to the contiguous activation of F2 and result in recurrent thromboembolism. Previous studies have reported three mutations (p.R596W, p.R596Q, and p.R596L) of *prothrombin* (coagulation factor II, F2) may lead to antithrombin resistance (ATR) and hereditary thrombosis (Miyawaki et al., 2012; Djordjevic et al., 2013; Sivasundar et al., 2013; Bulato et al., 2016; Tamura et al., 2017). In contrast, other mutations in *F2* may lead to bleeding diathesis (Kuijper et al., 2013; Takenouchi et al., 2019).

In this study, we enrolled a Chinese patient with recurrent venous thrombosis, coronary thrombosis and acute myocardial infarction. Whole exome sequencing was performed to detect the genetic lesion of the patient.

## Case Presentation

**FIGURE 1:**
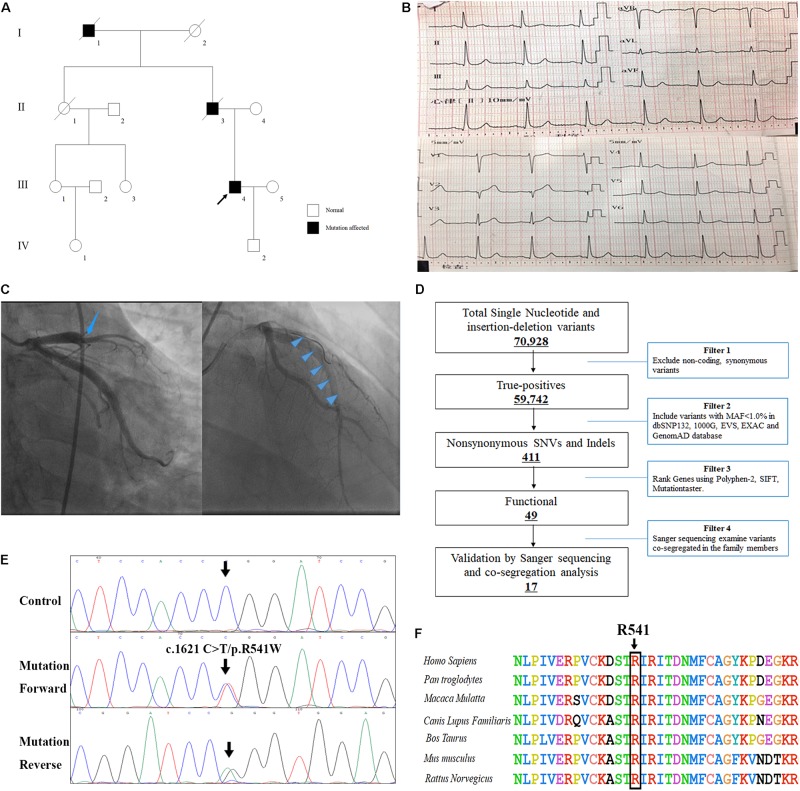
The clinical and genetic data of the patient with recurrent venous thrombosis, coronary thrombosis and acute myocardial infarction. **(A)** Pedigree of the family. Black circles/squares are affected, white are unaffected. Arrow indicates the proband. The ECG records **(B)** and coronary angiogram **(C)** of the proband. **(D)** Schematic representation of the filter strategies employed in our study. **(E)** Sanger DNA sequencing chromatogram demonstrates the heterozygosity for a *F2* missense mutation (c.1621 C>T/p.R541W). **(F)** Analysis of the mutation and protein domains of F2. The R541 affected amino acid locates in the highly conserved amino acid region in different mammals (from Ensembl). The black arrow shows the R541 site.

A Chinese patient with recurrent venous thrombosis, coronary thrombosis and acute myocardial infarction was enrolled from central south China (Figure 1A). The proband, a 37-year-old man, presented to our hospital because of repeated chest pain for 2 days, aggravated for 4 h. A medical history investigation showed that he had had three deep venous thromboses in the lower limbs and one portal vein thrombosis events during the past 10 years. His electrocardiogram showed Q wave elevation and slight ST segment elevation in lead V2 (Figure 1B). The coronary angiogram showed a total occlusion of the left anterior descending artery (Figure 1C). Ultrasound of the lower extremity venous and portal veins was normal, and right ventricular contrast echocardiography did not reveal a right to left shunt. Biochemical tests showed that activated partial thromboplastin time was decreased, accompanied by significantly increased troponin I and D-dimer levels. Other laboratory data are presented in Table 1. Anticardiolipin antibody was negative. The activity of protein C and antithrombin was normal, and the activity of protein S was slightly low (Table 1). The serum level of homocysteine was normal, and other secondary risk factors of thrombophilia were also ruled out, but the proband had a long history of smoking (10 years). Family history indicated that both his father (II-3) and grandfather (I-1) died from pulmonary thromboembolism. Finally, the patient was treated successfully by intracoronary thrombolysis, thrombus aspiration and balloon angioplasty (Figure 1C).

**TABLE 1:** The laboratory data of the proband.

	**Measure parameters**	**Data**	**Reference ranges**
Peripheral blood	White blood cells	5.42 × 10^9^/L	3.97–9.15 × 10^9^/L
	Red blood cells	4.06 × 10^12^/L	4.09–5.74 × 10^12^/L
	Hemoglobin	128 g/L	131–172 g/L
	Platelets	105 × 10^9^/L	85–303 × 10^9^/L
Blood chemistry	Na	145.2 mmol/L	137–147 mmol/L
	K	4.14 mmol/L	3.5–5.3 mmol/L
	Cl	107.3 mmol/L	99–110 mmol/L
	Total protein	52.2 g/L	65–85 g/L
	Aspartate aminotransferase	157.50 u/L	15–40 u/L
	Alanine aminotransferase	182.7 u/L	9–50u/L
	Blood urea nitrogen	3.04 mmol/L	1.7–8.3 mmol/L
	Creatinine	71 umol/L	40–100 umol/L
	Triglyceride	1.75 mmol/L	0.5–1.7 mmol/L
	HDL	0.64 mmol/L	0.8–1.9 mmol/L
	LDL	1.08 mmol/L	1.25–4.25 mmol/l
	Troponin I	26.557 ng/mL	0–0.034 μg/L
	HbA1c	4.9%	4.8%-6.0%
	C-reactive protein	8.23 mg/L	0–6 mg/L
Coagulation study	PT-INR	1.01	0.8–1.2
	Activated partial thromboplastin time	11.5S	25–34S
	Fibrinogen	2.34 g/L	2.0–4.0 g/L
	D-dimer	1.07 ug/mL	0–0.55 ug/mL
	Protein C activity	130%	60–140%
	Protein S activity	31.4%	63.5–149%
	Antithrombin	84%	83–128%
Anti-cardiolipin antibody	IgG	–	
	IgA	–	
	IgM	–	
Myocardial enzyme	Creatine kinase	629.1U/L	10–175 U/L
	Creatine kinase-MB fraction	81U/L	0–24 U/L

*PT-INR, prothrombin time international normalized ratio; LDL, low-density lipoprotein cholesterol; HDL, high-density lipoprotein cholesterol.*

## Laboratory Investigations

### Subjects

The study protocol was approved by the Review Board of the Hunan Provincial People’s Hospital and the study participants gave informed consent. Whole family members (one patient and eight healthy members) were enrolled and diagnosed by electrocardiogram, coronary angiogram and laboratory inspection.

### Whole Exome Sequencing

Genomic DNA was extracted from peripheral blood lymphocytes of all subjects by using the JetFlex^TM^ Genomic DNA Purification Kit (Invitrogen^TM^). The proband was chosen for the whole exome sequencing at the Kindstar Global Company (Wuhan, China). Agilent SureSelect Human All Exon V6 kits was applied to capture the exomes and the sequencing platform was an Illumina HiSeq X-10. The strategies for data filtering referred to Figure 1 as we previous described (Fan et al., 2019).

### Mutation Validation and Co-segregation Analysis

After the filtering process, all mutations found for the patient were validated by Sanger sequencing. The primer pairs used for PCR amplification were designed with Primer 3 (primer sequences will be provided upon request). The sequences of the PCR products were determined using the ABI 3100 Genetic Analyzer (ABI, Foster City, CA, United States).

Whole-exome sequencing yielded 9.21 Gb of data with 99.7% coverage of target regions, and 99.0% of the target regions were covered over 10 fold. After alignment and single nucleotide variant calling, 70,928 variants were identified in the proband. We then performed data filtering as shown in Figure 1D and 17 variants were retained after co-segregation analysis (Table 2). Further, we analyzed the inheritance pattern, OMIM clinical phenotypes, ToppGene function and American College of Medical Genetics classification of these 17 gene variants (Table 2). The mutation c.1621 C>T/p.R541W of *F2* was highly suspected to be the genetic lesion of the patient (Figure 1E). The mutation identified in the proband was absent in all available healthy relatives included in this study, but unfortunately it was not tested in the two deceased symptomatic relatives (father and paternal grandfather). This new mutation (p.R541W), resulting in a substitution of arginine by tryptophan, was located in a highly evolutionarily conserved site (Figure 1F) and was also absent in our 200 local control subjects (Fan et al., 2018). A previous *in vitro* study proved that this mutation (p.R541W) may substantially impaire inactivation by antithrombin, resulting in a prolonged clotting function and leading to an ATR phenotype, which further proved that the mutation may be a high risk factor for thrombosis (Tamura et al., 2017).

**TABLE 2:** The mutations list after co-segregation analysis.

**CHR**	**POS**	**RB**	**AB**	**Gene**	**Mutation**	**MAF in gnomAD**	**SIFT**	**PolyPhen-2**	**Mutation taster**	**OMIM clinical phenotype**	**ToppGene function**	**ACMG classification**
2	17922901	GT	G	SMC6	NM_024624: c.215delA:p.N72fs	0	–	–	1,D	–	Positive regulation of chromosome segregation	PM4
3	167183012	A	G	SERPINI2	NM_006217: c.T848C:p.V283A	0	0.008,D	0.999,D	0.98,D	–	Endopeptidase regulator activity	PP3
3	183951380	G	A	VWA5B2	NM_138345: c.G547A:p.G183R	0.00004	0.044,D	1.0,D	1,D	–	Core extracellular matrix	PP3
4	123147863	T	C	KIAA1109	NM_015312: c.T2795C:p.L932P	0	0.002,D	0.971,D	1,D	AR: Alkuraya- Kucinskas syndrome	Adipose tissue development	BP5
6	76744394	A	G	IMPG1	NM_001282368: c.T178C:p.F60L	0.00003	0.022,D	0.877,P	0.95,D	AD: Macular dystrophy	Extracellular matrix structural constituent	BP5
8	144887433	C	T	SCRIB	NM_015356: c.G2519A:p.R840Q	0.00004	0.009,D	1.0,D	0.99,D	–	Synaptic vesicle targeting	PP3
10	55779951	C	G	PCDH15	NM_001142763: c.2766+1G>C	0	–	–	1,D	AR: Usher syndrome	Auditory receptor cell stereocilium organization	BP7
11	46751078	C	T	F2	NM_000506: c.C1621T:p.R541W	0	–	0.98,D	0.97,D	AR: Hypoprothrom- binemia; AD: Thrombophilia	Thrombospondin receptor activity	PS3
11	129743816	G	A	NFRKB	NM_006165: c.C2449T:p.R817W	0.0001	0.0,D	1.0,D	0.99,D	–	Protease binding	PP3
12	41331471	G	A	CNTN1	NM_175038: c.G1177A:p.A393T	0	0.0,D	1.0,D	1,D	AR: Myopathy	Anchored component of membrane	BP5
12	56349931	A	G	PMEL	NM_001200053: c.T1169C:p.V390A	0	0.002,D	0.894,P	1,D	–	Positive regulation of melanin biosynthetic process	PP3
13	76140903	T	A	UCHL3	NM_001270952: c.T148A:p.F50I	0	0.001,D	0.998,D	1,D	–	Positive regulation of fat cell differentiation	PP3
15	62148523	G	A	VPS13C	NM_017684: c.C10909T: p.P3637S	0	0.021,D	1.0,D	0.99,D	AR: Parkinson disease	Regulation of autophagy of mitochondrion	BP5
15	90192287	C	T	KIF7	NM_198525: c.G841A:p.A281T	0.00001	0.004,D	0.897,P	0.99,D	AR: Joubert syndrome	Regulation of smoothened signaling pathway	BP5
16	67980481	A	G	SLC12A4	NM_001145962: c.T2303C:p.V768A	0	0.016,D	0.99,D	1,D	–	Chloride symporter activity	PP3
18	44113218	G	C	LOXHD1	NM_001308013: c.C661G:p.R221G	0.00001	0.003,D	0.999,D	0.99,D	AR: Deafness	Calcium channel activity	BP5
19	48807321	G	A	CCDC114	NM_144577: c.C631T:p.Q211X	0	–	–	–	AR: Ciliary dyskinesia	Outer dynein arm assembly	BP5

*CHR, chromosome; POS, position; RB, reference sequence base; AB, alternative base identified; MAF, minor allele frequency; D, damaging; P, probably damaging; AR, autosomal recessive; BP, Benign Supporting; PP, pathogenicity supporting; PS, pathogenicity strong.*

## Discussion

In the HGMD database, 70 mutations of *F2* have been identified in patients. However, only three mutations in one site (R596) were identified in patients with deep venous thrombosis (Miyawaki et al., 2012; Djordjevic et al., 2013; Sivasundar et al., 2013; Bulato et al., 2016). The mutation Belgrade (p.R596Q) of *F2* can lead to pulmonary embolism, acute mesenteric vein thrombosis and myocardial infarction (Djordjevic et al., 2013). Other mutations are related to dysprothrombinemia and prothrombin deficiency (Pasmant et al., 2011). In the present study, we identified a novel mutation (c.1621 C>T/p.R541W) of *F2* in a patient with recurrent venous thrombosis, coronary thrombosis and acute myocardial infarction. A previous *in vitro* study proved that this mutation (p.R541W) may lead to ATR and increase the risk of thrombosis (Tamura et al., 2017). Our study may be the second report that a mutation of *F2* may also lead to coronary thrombosis and acute myocardial infarction.

Coagulation factor II is proteolytically cleaved to form thrombin in the first step of the coagulation cascade which ultimately results in the stemming of blood loss (Friedmann et al., 2019). F2 also plays a role in maintaining vascular integrity during development and postnatal life (Sun et al., 2002). The site p.R541 of *F2* together with T540, E592, and K599 compose the sodium binding region in conjunction with R596 (Rouy et al., 2006). The disruption of the sodium binding region of thrombin induces resistance to antithrombin inactivation (Tsiang et al., 1996; Dang et al., 1997). Mutations at p.R541 may lead to an ATR-phenotype because R541 in thrombin associates with antithrombin E264 via water mediated H-bonds and connects to a neighboring residue (T540) through the nitrogen atom of the peptide bond (Virno et al., 2007; Tamura et al., 2017). Therefore, amino acid substitution at this residue may result in resistance to antithrombin inactivation and increase the risk of thrombosis.

Previously, researchers developed the residual clotting activity (RCA) assay after 30 min inactivation with antithrombin to estimate the thrombosis risk of the mutations *in vitro*. The RAC score of p.R541W was 2.49 while the wild type score was 1.00, which indicated that this mutation (p.R541W) showed an ATR phenotype and may lead to thrombosis (Tamura et al., 2017). In our patient, D-dimer, the biomarker of thrombosis, was obviously elevated (Wells et al., 2003). The shortened activated partial thromboplastin time indicated an increased tendency to form thrombus (Mina et al., 2010). The elevated troponin I suggested that the patient suffered from acute myocardial infarction (Reichlin et al., 2011). Both *in vitro* studies and laboratory investigations indicated that the mutation (p.R541W) of *F2* is the genetic lesion (potential etiology) of the patient.

Interestingly, *F2* mutations associated with ATR can have pathogenic effects on different territories. Mutations (p.R596W and p.R596L) lead to vein thrombosis and pulmonary embolism. The mutation Belgrade (p.R596Q) displayed pulmonary embolism, acute mesenteric vein thrombosis and myocardial infarction (Miyawaki et al., 2012; Sivasundar et al., 2013; Bulato et al., 2016). In our study, we found that the mutation p.R541W resulted in venous thrombosis, coronary thrombosis and acute myocardial infarction. The mutation we have identified here may prevent F2 from being inhibited by antithrombin, which will lead to the constitutive activation of F2 and result in recurrent thromboembolism. We believe that p.R541W is a prothrombotic mutation, and the localization of the thrombus will probably depend on additional risk factors. Cardiovascular risk factors may trigger a thrombotic event in the arterial territory. For example, the proband had a 10-year smoking history, which is an important risk factor for cardiovascular disease (Govender et al., 2019) and may lead to coronary thrombosis together with the F2 mutation p.R541W. In addition, we also found another 16 unique polymorphism sites (Table 2). According to the inheritance model, eight polymorphism sites that were autosomal recessive were excluded. At another nine polymorphism sites, only the polymorphism site p.R840Q of *SCRIB* may be related to cardiovascular risk factors. Previous studies have proved that SCRIB is required for the morphogenesis of the ventricular myocardium (Boczonadi et al., 2014). This unique SNP may also be a cardiovascular risk factor and lead to coronary thrombosis together with the F2 mutation (p.R541W). In short, the p.R541W is a prothrombotic mutation, and other cardiovascular risk factors may also have effects on the localization of the thrombus.

In addition, many people with thrombophilia do not have symptoms and never have health problems seen in the clinic (Lim and Moll, 2015; Stevens et al., 2016). These characteristics of thrombophilia may explain the spectrum of disease of p.T540S and p.R541Q of *F2*, similar sites to p.R541W, in the dbSNP database^1^. Meanwhile, both minor allele frequencies of rs752961431 (p.T540S) and rs552953108 (p.R541Q) in the EXAC, GnomAD and 1000 Genomes databases were all far less than 0.001. An *in vitro* study suggested that both p.T540S and p.R541Q of *F2* also showed an ATR phenotype (Tamura et al., 2017). To further understand these differences, more clinical data and functional experiments might be required. For example, in this study, we identified the p.R541W mutation in a patient with recurrent venous thrombosis, coronary thrombosis and acute myocardial infarction. Taken together with our clinical genetics data and previous *in vitro* studies, this study may confirm that the site p.R541 may play a crucial role in affecting thrombin-antithrombin complex formation and/or stabilization.

In summary, we identified a novel mutation (c.1621 C>T/p.R541W) of *F2* in a Chinese patient with recurrent venous thrombosis, coronary thrombosis and acute myocardial infarction. The present study was consistent with a previous *in vitro* study showing that the mutation may result in resistance to antithrombin inactivation and increase the risk of thrombosis. This may be the second report of a mutation in *F2* can lead to coronary thrombosis and acute myocardial infarction worldwide. Our study not only expand the spectrum of *F2* mutations associated with ATR and will contribute to the genetic diagnosis and counseling of families with thrombosis but also provides new insight into the role of *F2* mutations in coronary thrombosis.

## Data Availability Statement

The datasets used and/or analyzed during the current study are available from the corresponding author upon reasonable request.

## Ethics Statement

Written informed consent was obtained from the parents. The study was approved by the Ethics Committee of Hunan Provincial People’s Hospital, Hunan, China and performed in accordance with the principles enshrined in the Declaration of Helsinki. The patients/participants provided their written informed consent to participate in this study.

## Author Contributions

YT and LZ enrolled the patients and performed the genetic analysis. JJ did the PCR. WX, YL, MD, ZL, HP, and YZ assisted in diagnosis. ZZ and L-LF wrote the draft and supported the study.

## Conflict of Interest

The authors declare that the research was conducted in the absence of any commercial or financial relationships that could be construed as a potential conflict of interest.

## Footnotes

1 https://www.ncbi.nlm.nih.gov/snp/

## Abbreviations

EXAC: The Exome Aggregation Consortium
F2: coagulation factor II
HGMD: The Human Gene Mutation Database
OMIM: Online Mendelian Inheritance in Man
PCR: polymerase chain reaction
SNP: single nucleotide polymorphisms.
